# Diagnostic Accuracy of Clinical Examination and Imaging Findings for Identifying Subacromial Pain

**DOI:** 10.1371/journal.pone.0167738

**Published:** 2016-12-09

**Authors:** Angela Cadogan, Peter J. McNair, Mark Laslett, Wayne A. Hing

**Affiliations:** 1 Health and Rehabilitation Research Institute, AUT University, Auckland, New Zealand; 2 Faculty of Health Sciences and Medicine, Bond University, Robina, Queensland, Australia; Kanazawa University, JAPAN

## Abstract

**Background:**

The diagnosis of subacromial pathology is limited by the poor accuracy of clinical tests for specific pathologies. The aim of this study was to estimate the diagnostic accuracy of clinical examination and imaging features for identifying subacromial pain (SAP) defined by a positive response to diagnostic injection, and to evaluate the influence of imaging findings on the clinical diagnosis of SAP.

**Methods and Findings:**

In a prospective, diagnostic accuracy design, 208 consecutive patients presenting to their primary healthcare practitioner for the first time with a new episode of shoulder pain were recruited. All participants underwent a standardized clinical examination, shoulder x-ray series and diagnostic ultrasound scan. Results were compared with the response to a diagnostic block of xylocaine^TM^ injected into the SAB under ultrasound guidance using ≥80% post-injection reduction in pain intensity as the positive anaesthetic response (PAR) criterion. Diagnostic accuracy statistics were calculated for combinations of clinical and imaging variables demonstrating the highest likelihood of a PAR. A PAR was reported by 34% of participants. In participants with no loss of passive external rotation, combinations of three clinical variables (anterior shoulder pain, strain injury, absence of symptoms at end-range external rotation (in abduction)) demonstrated 100% specificity for a PAR when all three were positive (LR+ infinity; 95%CI 2.9, infinity). A full-thickness supraspinatus tear on ultrasound increased the likelihood of a PAR irrespective of age (specificity 98% (95%CI 94, 100); LR+ 6.2; 95% CI 1.5, 25.7)). Imaging did not improve the ability to rule-out a PAR.

**Conclusion:**

Combinations of clinical examination findings and a full-thickness supraspinatus tear on ultrasound scan can help confirm, but not exclude, the presence of subacromial pain. Other imaging findings were of limited value for diagnosing SAP.

## Introduction

Shoulder pain is a common complaint in primary health care resulting in significant pain and disability, loss of productivity and health care costs [1]. The lifetime prevalence of shoulder pain in the general population is reported between 10% to 67% [2], and up to 6% of the population consult their general practitioner annually with an episode of shoulder pain [3, 4].

Subacromial disorders are the most common disorders accounting for up to 85% of shoulder conditions seen in primary care [5, 6]. Pain in the subacromial region can be caused by a number of pathological conditions including subacromial bursitis, rotator cuff tendinosis, calcific tendinosis and rotator cuff tears. There is a growing body of evidence for the specific management of these subacromial conditions including corticosteroid injections for pain relief [7], image-guided fenestration of calcific deposits [8, 9], physiotherapy and specific strengthening for non-calcific rotator cuff tendinosis and small rotator cuff tears [10–12] and surgery for large rotator cuff tears or lack of response to non-surgical measures [13]. Whether appropriate management interventions are applied is dependent upon the accurate diagnosis of the condition in the first instance [14].

In clinical practice, examination of the painful shoulder pain is typically aimed at identifying a specific pathoanatomic diagnosis to inform selection of appropriate treatment interventions [15]. This diagnostic approach relies upon the availability of clinical tests with high levels of accuracy for specific shoulder pathology. Many tests commonly used in clinical practice are reported to be diagnostic of specific pathologies including the various stages of impingement pathology (bursitis, partial and full thickness tears) [16, 17], large rotator cuff tears [18], superior labral tears [19, 20], posterior labral tears [21] and acromioclavicular joint pathology [19, 22]. However, it has been consistently demonstrated that commonly used clinical tests lack accuracy for specific pathoanatomic lesions of the shoulder when compared with imaging or surgical reference standards [15, 23, 24]. Previous diagnostic accuracy studies have shown that physical examination tests lack accuracy for, and are not able to differentiate between specific shoulder anatomic structures [15, 24, 25], or between specific pathologies of the rotator cuff (tendinosis versus a tear) [17, 26] and the glenohumeral joint (capsular versus labral pathology) [26]. One possible explanation for the poor accuracy of clinical tests lies in the complex anatomic and functional relationship between intra- and extra-articular shoulder structures making it difficult to isolate specific structures during clinical tests resulting in similar clinical presentations of different shoulder disorders.

The increasing use of diagnostic imaging such as x-ray and diagnostic ultrasound scans for shoulder pathology in primary health care settings may be related to low levels of practitioner confidence in making an accurate clinical diagnosis [27, 28]. While imaging investigations may provide evidence of pathological tissue changes, the high prevalence of asymptomatic pathology identified on imaging, particularly in ageing populations complicates the interpretation of imaging results with respect to symptomatic relevance [29–31]. This may result in the application of inappropriate treatment interventions if clinicians ascribe symptomatic causation to the reported imaging findings in the absence of clinical correlation. The majority of previous diagnostic accuracy studies used imaging or surgical visualisation of shoulder pathology as the reference standard test on the assumption that the visualised pathology was the source of pain. Asymptomatic shoulder pathology is common, and test accuracy, especially specificity values, are confounded in this design. This may provide another explanation for the poor reported accuracy of clinical tests in previous studies that used imaging or surgical visualization of shoulder pathology as the reference standard test.

Evidence is now clear that the pursuit of an accurate pathoanatomic diagnosis of shoulder pain using clinical tests alone is not possible in the primary care environment. Even when radiological imaging investigations are available, the high prevalence of asymptomatic pathology on imaging complicates the interpretation of imaging with respect to symptoms. The problems associated with the pathoanatomic approach to the clinical diagnosis of shoulder pain in primary care may lead the practitioner to an inaccurate diagnosis and/or inappropriate treatment interventions that may adversely affect patient outcome and result in inappropriate use of healthcare resources.

Instead of attempting to identify specific pathologies using clinical tests, the ability to first differentiate the source of the patients’ symptoms as arising from either the subacromial region, the glenohumeral joint or the acromioclavicular joint may be a more realistic clinical endeavour in the primary care setting. This would provide a sound basis from which to make clinical decisions regarding subsequent investigations and management. Having identified a primary subacromial pain source, additional imaging investigations could then be selectively and appropriately applied to further differentiate specific subacromial pathology where this may alter management decisions. The ability to clinically identify the likely pain source prior to diagnostic imaging investigations would also enhance the ability to interpret the symptomatic relevance of subsequent imaging findings, and facilitate the application of, or referral for appropriate treatment interventions for specific subacromial conditions such as fenestration of calcific deposits, physiotherapy for rotator cuff tendinosis or orthopaedic referral for acute, large rotator cuff tears.

Diagnostic injection of local anaesthetic (diagnostic block) is the accepted reference standard for identification of the tissue source of musculoskeletal pain [32, 33], with a marked reduction in, or complete abolition of post-injection pain being indicative of a positive response. Neer [34] described the ‘subacromial injection test’ for subacromial impingement pain [34, 35] using injection of local anaesthetic into the subacromial space to differentiate subacromial pain from other sources of shoulder pain and subacromial injections have since become a widely used diagnostic tool for painful shoulder conditions [36, 37]. While diagnostic injections into the subacromial space provide an accurate method for confirming the subacromial region as the source of symptoms, it is not clinically feasible to perform these invasive procedures on all patients. Injections also fall outside the scope of practice for many primary healthcare practitioners precluding their widespread use. The ability to identify clinical tests that accurately predict the response to subacromial diagnostic injection may provide a “proxy” for the subacromial injection test that would help the clinician to confirm or exclude a primary subacromial pain source without the need for invasive diagnostic injection procedures.

Little attention has been paid to identification of clinical examination features with the ability to predict the response to subacromial diagnostic injection. Previous analysis of our clinical examination data using stepwise regression analyses identified anterior shoulder pain, history of injury and the absence of symptoms in external rotation as the strongest predictors of a positive response following injection of local anaesthetic into the subacromial space [38]. However, stepwise regression analysis is known to be adversely affected by variable collinearity and coefficient bias [39, 40], and variable selection methods may fail to recognise variables that are of clinical and practical significance. Whether these same variables demonstrate higher diagnostic probability or likelihood than other clinical variables for accurately identifying subacromial pain has not been evaluated. In addition, despite the widespread use of diagnostic imaging in clinical practice, we are not aware of any other studies that have investigated the symptomatic relevance of specific imaging findings such as subacromial bursitis and rotator cuff tears in patients with subacromial pain by comparing them with the results of a diagnostic local anaesthetic injection.

The aim of this study was to identify individual, and combinations of clinical examination and imaging features with the highest levels of diagnostic accuracy for identifying subacromial pain (SAP) defined by a positive response to a diagnostic injection of local anaesthetic into the subacromial space, and estimate the influence of imaging findings on the ability to accurately identify SAP when combined with clinical findings.

## Methods

In a prospective, diagnostic validity design, the results of clinical examination and diagnostic imaging tests (index tests) were compared with the results of a diagnostic injection of local anaesthetic into the subacromial space (reference standard procedure) to identify pain of subacromial origin. The study was designed according to existing guidelines for design of diagnostic accuracy studies [41]. Ethical approval was granted by the New Zealand Ministry of Health (Upper South) Ethics Committee and all participants provided written informed consent.

### Participants

Participants were recruited consecutively from community-based medical and physiotherapy practices across Christchurch, New Zealand. Patients over the age of 18 years, presenting to their general practitioner or physiotherapist for the first time with a new episode of shoulder pain and with the ability to follow verbal instructions were eligible for inclusion in the study. Exclusion criteria were known fractures or dislocations around the shoulder complex, referred pain from the cervical spine, sensory or motor deficit involving the upper limb, previous surgery to the shoulder or cervical spine, or contraindications to study procedures.

### Procedures

#### Clinical examination

All included participants received a standardized clinical examination including medical history, symptom chart, patient history and physical examination. Details were collected regarding the mechanism of pain onset and pain location (S1 Table). The physical examination consisted of the following tests that are used in the clinical diagnosis of subacromial conditions: assessment of passive glenohumeral joint range of motion (external rotation in neutral and 90 degrees of abduction) [42], resisted rotator cuff tests and orthopaedic tests performed as originally described; Hawkins-Kennedy test [43], drop-arm test [42], empty can test [44], external rotation lag sign [18].

**Fig 1:**
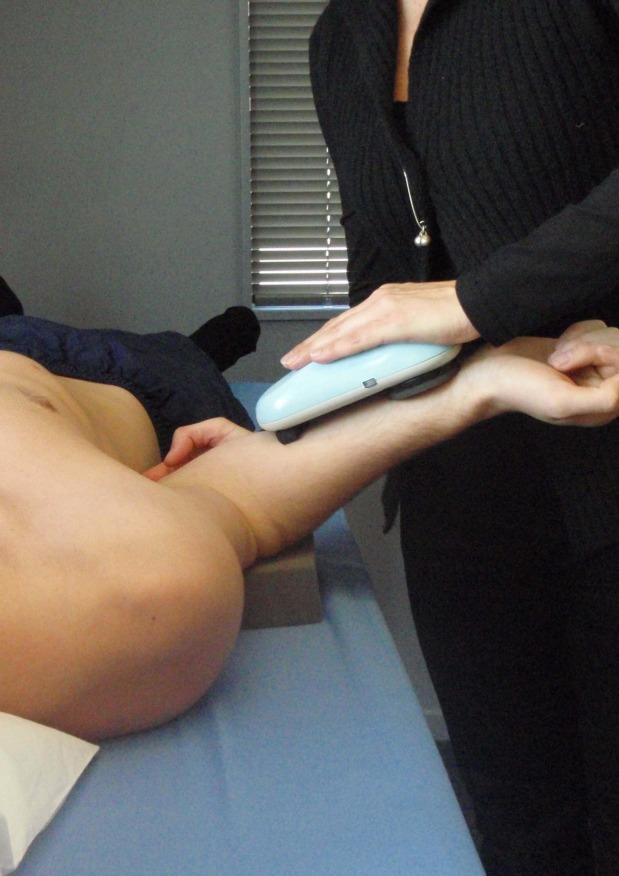
Measurement of passive external rotation. Method used to measure passive range of motion in shoulder external rotation using a hand-held dynamometer (Industrial Research Ltd, Christchurch, New Zealand) with the ability to standardize force-overpressure at end range of motion.

During the physical examination, those tests provocative of typical pain were identified for use in pre- and post-injection testing. Indeterminate results of clinical examination tests were recorded and coded as missing data. Passive range of motion was recorded using a hand-held dynamometer (Industrial Research Ltd, Christchurch, New Zealand) with the ability to standardize force overpressure at end-range of motion (Fig 1). Procedures for passive range of motion and strength testing have been described in detail elsewhere [45]. Intraobserver reliability for passive range of motion tests was subsequently evaluated with 95% limits of agreement within 10° [46] and intraclass correlation coefficients exceeding 0.80 for all tests. A passive external rotation deficit of ≥30 degrees compared with the unaffected side was used as the cut-point for the external rotation deficit variable. This value is consistently used to classify capsular restriction of glenohumeral joint range of motion associated with frozen shoulder and glenohumeral arthritis [47, 48]. The clinical examination was conducted by an experienced musculoskeletal clinician with 22 years of experience.

#### X-ray and Diagnostic Ultrasound Scan

Within one week of the clinical examination, participants underwent a standardized series of shoulder radiographs (x-ray) consisting of anterior-posterior (AP) views in neutral, external and internal rotation, axial view and outlet view [49]. X-rays were reported by experienced musculoskeletal radiologists using a standardized report form that included specific abnormalities of the ACJ, acromion, GHJ and calcific deposits. Imaging diagnostic criteria are presented in Table 1.

**Table 1:** Radiologic diagnostic criteria.

Pathology	Imaging Diagnostic Criteria
**X-Ray**	
Acromioclavicular joint:	
- arthropathy/degenerative change	joint space narrowing, subchondral sclerosis, subchondral cystic change or marginal osteophytes.
- Osteolysis	bony resorbtion or increased lucency in distal clavicle or acromion.
Glenohumeral joint:	
- arthropathy/degenerative change	joint space narrowing, subchondral sclerosis, subchondral cystic change or marginal osteophytes.
- other	loose bodies, joint calcifications.
Calcification of rotator cuff components:	calcific deposits with measurable dimensions only. Does not include ‘flecks’ of calcium.
- supraspinatus	calcific deposits adjacent to the greater tuberosity on AP-external rotation x-ray view.
- infraspinatus	calcific deposits adjacent to the greater tuberosity on AP-internal rotation x-ray view.
- subscapularis	calcific deposits in the anterior shoulder region on axial x-ray view.
**Ultrasound**^a^	
ACJ pathology	Capsular hypertrophy, cortical irregularity or osteophytes, capsular bulge, joint space narrowing or widening.
Glenohumeral joint effusion	more than 2mm between posterior glenoid labrum and posterior capsule.
Rotator cuff:	
- normal	normal contour, normal echogenicity.
- calcification	focal increase in echogenicity with or without shadowing.
- tendinosis	Rotator cuff or LHB: tendon thickening or decreased echogenicity.
- intrasubstance tear	hypoechoic change not extending to articular or bursal surface.
- partial thickness tear	SSp and ISp: hypoechoic change extending to either the articular or bursal surface. Subscapularis: partial fibre discontinuity.
- full thickness tear	SSp and ISp: hypoechoic region extends from bursal to articular surface. Subscapularis: complete fibre discontinuity.
Subacromial bursa:	
- bursitis	hypoechoic fluid or effusion present and >2mm thick.
- bursal thickening	≥2mm measured from deep margin of deltoid to superficial margin of supraspinatus.
- “bunching”	Fluid distension of the SAB or ‘buckling’ of the rotator cuff during abduction

*Abbreviations*: AP, antero-posterior view; ACJ, acromioclavicular joint; LHB, long head of biceps tendon; SSp, supraspinatus; ISp, infraspinatus; SAB, subacromial bursa

^a^ Definitions based upon accepted diagnostic criteria [50, 51]

Diagnostic ultrasound scans were performed by trained and experienced musculoskeletal sonographers and reported by fellowship trained musculoskeletal radiologists. Examinations were performed using a Philips IU22 machine with a 5–12MHz linear array probe using a standardized scan procedure: [50, 52] 1) patient sitting with palm face up on their knee (long head of biceps tendon); 2) elbow tucked into their side with external rotation of the shoulder (subscapularis); 3) arm resting on lap in neutral rotation with elbow behind body (supraspinatus); 4) hand in the small of the back with palm facing outwards to visualize (supraspinatus); 5) hand placed on the opposite shoulder (infraspinatus, ACJ, posterior labrum and glenohumeral joint). Scanning was conducted along the line of each tendon and at 90 degrees to the tendon.

The subacromial bursa was observed during dynamic abduction and ‘bunching’ under the acromion was recorded. Subacromial bursal dimensions were measured from the deep margin of deltoid muscle to superficial margin of supraspinatus tendon in all cases where this distance was measurable (dimensions exceeding 1mm).

#### Reference Standard Procedure

**Fig 2:**
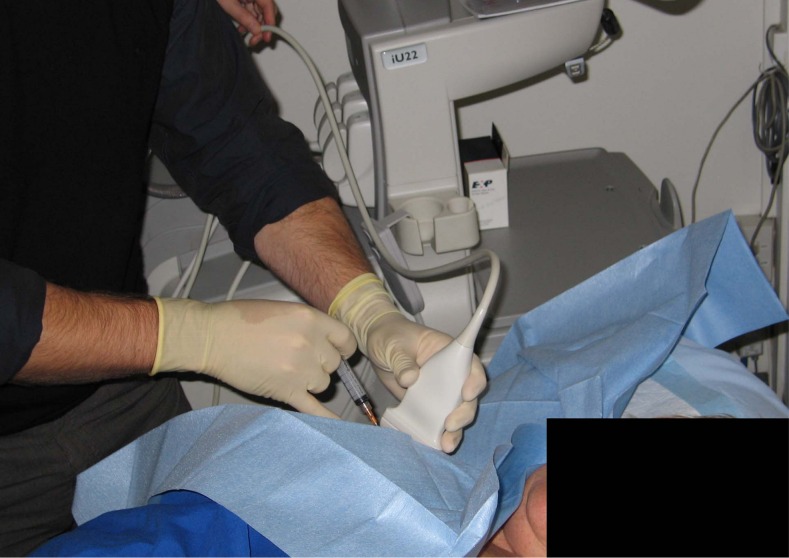
Ultrasound guided diagnostic injection into the subacromial bursa

An ultrasound-guided injection of local anaesthetic into the subacromial bursa was used as the reference standard procedure (Fig 2). Participants were positioned supine with the arm in external rotation. Under aseptic conditions, a 22-gauge needle was used to inject 5mL of 1% lidocaine hydrochloride (xylocaine^TM^) into the SAB under ultrasound guidance using an anterior approach. When needle placement inside the subacromial bursa was confirmed by ultrasound, the contents of the syringe were emptied into the bursa. All injections were performed by a fellowship trained musculoskeletal radiologist.

Immediately prior to the injection, all participants were examined using up to six tests that had previously been identified during the clinical examination as being provocative of their typical symptoms. Pre-injection pain intensity was then recorded for each clinical test on a 100mm visual analogue scale (VAS) where 0mm indicated “no pain” and 100mm represented “worst imaginable pain”. Tests were repeated between 5 and 15 minutes following the diagnostic block and post-injection pain intensity VAS scores recorded again. The average change in pain intensity from all clinical tests was then calculated. A positive anaesthetic response was determined by 80% or more post-injection reduction in pain intensity (80% PAR). This is similar to the criteria for PAR used in other studies involving diagnostic blocks [53–55] and represents a high level of confidence that the target structure is a major contributor to symptoms.

The investigator performing the clinical examination and pre- and post-injection clinical tests was blinded to any diagnostic or treatment information from referring practitioners and to the results of imaging investigations. The radiologist who performed the SAB diagnostic block was blinded to any clinical information including the results of pre-injection provocative clinical testing.

### Data Analysis

Sample size was estimated using methods for estimates for diagnostic accuracy studies described by Flahault et al. with the minimal acceptable lower confidence limit set at 0.75 and expected sensitivity/specificity both set at 0.90, with adjustment following sub-group analysis of the first 100 cases to maintain precision of confidence interval estimates [56].

Cross-tabulations of clinical examination and imaging results with an 80% PAR was carried out using Statistical Package for the Social Sciences (SPSS) version 22.0 (IBM Corporation®).[57] Diagnostic accuracy characteristics including sensitivity, specificity, predictive values, positive likelihood ratios (+LR) and negative likelihood ratios (-LR) and diagnostic odds ratios (DOR) with 95% confidence intervals (CI) were calculated for individual clinical examination and imaging variables for an 80% PAR following SAB diagnostic injection using Confidence Interval Analysis software [58]. Variables with the most favourable diagnostic characteristics based upon likelihood ratios were then combined and diagnostic characteristics re-calculated. Due to the well documented increase in rotator cuff tear prevalence in older populations, variable combinations were stratified across two age-groups using 50 years as the cut-point [29].

## Results

**Fig 3:**
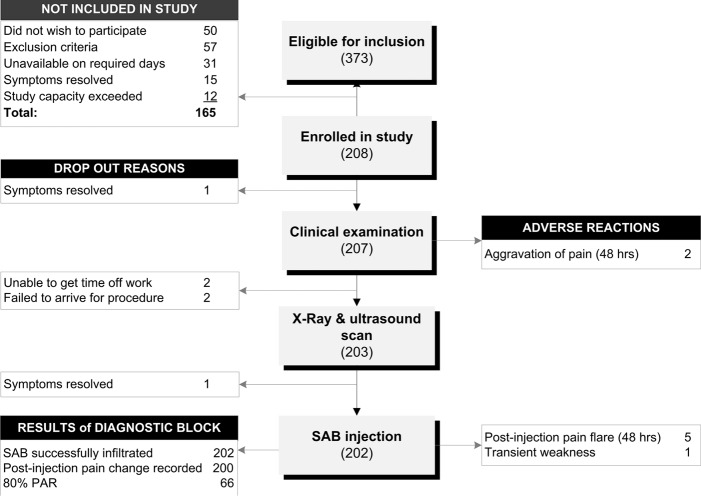
Flow chart of participants through the study.

Three hundred and seventy three patients were referred to the study between July 2009 and June 2010 resulting in 208 participants being included in the study (Fig 3). Those excluded from the study reported shorter duration of symptoms (2 weeks; IQ range 4 weeks; p<0.001). Participant characteristics are presented in Table 2. No demographic variables were associated with a PAR (p>0.05). Frequency distributions for clinical examination variables in the PAR and negative anaesthetic response (NAR) groups are provided in the supplementary file (S1 Table). Missing or indeterminate data exceeded 5% for painful arc in abduction (11% had insufficient abduction range of motion to complete the test). Frequency distributions for imaging variables are presented in Table 3. Those aged over 50 years had a higher prevalence of full-thickness supraspinatus tears (13%) compared with those younger than 50 years (2%) (OR 9.7, 95%CI 2.0, 48.3; p = 0.001).

**Table 2:** Participant characteristics.

Characteristics	All participants		PAR Group	NAR Group	p-value
	(N = 202)		(n = 69)	(n = 133)	
	Mean (SD)	Range	Mean (SD)	Mean (SD)	
Age (years)	42 (14)	18–81	42 (12)	42 (15)	0.834
Height (cm)	172 (10)	147–199	171 (9)	172 (10)	0.120
Weight (kg)	80.6 (18.0)	50.3–189.0	80.2 (21)	81 (17)	0.883
Symptom duration (weeks)^†^	7 (13)^†^	0–175	7 (14) ^†^	7 (12) ^†^	0.851
VAS (worst)	62 (23)	3–100	62 (22)	64 (24)	0.556
VAS (average)	37 (22)	1–100	36 (18)	37 (23)	0.798
SPADI pain score (%)	50 (22)	0–100	50 (21)	51 (22)	0.924
SPADI disability score (%)	30 (23)	0–96	28 (22)	31 (22)	0.455
SPADI total (%)	38 (21)	0–98	36 (20)	39 (21)	0.639
Male gender	102 (51%)		31 (47%)	71 (55%)	0.365
Right hand dominant	171 (87%)		58 (88%)	113 (87%)	1.00
Dominant arm affected	103 (53%)		34 (52%)	69 (53%)	0.880
In paid employment	158 (80%)		54 (82%)	104 (80%)	0.850
Off work	6 (3%)		0 (0%)	6 (5%)	0.099
Co-existent medical conditions	66 (34%)		21 (32%)	45 (35%)	0.751
Smoker	37 (19%)		12 (19%)	25 (19%)	1.00

*Abbreviations*: PAR, positive anaesthetic response (≥80% post-injection reduction in pain intensity); NAR, negative anaesthetic response (<80% reduction in post-injection pain intensity); VAS, 100mm visual analogue pain score in previous 48 hours; SPADI, Shoulder Pain & Disability Index; ACC, the Accident Compensation Corporation.

^†^ Variable not normally distributed; median (interquartile range) are presented.

**Table 3:** Prevalence of imaged pathology in those reporting a positive and negative anaesthetic response to diagnostic injection into the subacromial bursa (N = 196).

Pathology identified		PAR group	NAR group	OR	p-value
		(n = 66)	(n = 130)		
	N	n (%)	n (%)	(95%CI)	
**X-ray**					
Any ACJ pathology	32	8 (12)	24 (19)	0.6 (0.3, 1.5)	0.312
ACJ arthropathy	24	6 (9)	18 (14)	0.6 (0.2, 1.7)	0.489
ACJ osteolysis	7	1 (2)	6 (5)	0.3 (0.0, 2.7)	0.428
GHJ pathology	10	2 (3)	8 (6)	0.5 (0.1, 2.3)	0.500
Supraspinatus calcification	16	9 (14)	7 (5)	2.8 (1.0, 8.0)*	0.050
Infraspinatus calcification	7	2 (3)	5 (4)	0.8 (0.2, 4.2)	1.000
Subscapularis calcification	6	0 (0)	6 (5)	1.5 (1.4, 1.7)	0.181
**Ultrasound**					
SAB pathology^†^	135	46 (70)	89 (69)	1.1 (0.6, 2.0)	1.000
Bursal bunching (acromion)	81	30 (46)	51 (42)	1.2 (0.6, 2.2)	0.645
Supraspinatus tear (any)	45	18 (27)	27 (21)	1.4 (0.7, 2.8)	0.369
- intrasubstance tear	23	9 (14)	14 (11)	1.3 (0.5, 3.2)	0.640
- PTT bursal surface	4	0 (0)	4 (3)	1.5 (1.4, 1.7)	0.302
- PTT articular surface	8	2 (3)	6 (5)	0.6 (0.1, 3.3)	0.720
- FTT	10	7 (11)	3 (2)	5.0 (1.3, 20.1)*	0.033
Infraspinatus tear (any)	3	1 (2)	2 (2)	1.0 (0.1, 11.1)	1.000
- PTT	1	0 (0)	1 (1)	1.5 (1.4, 1.7)	1.000
- FTT	1	0 (0)	1 (1)	1.5 (1.4, 1.7)	1.000
Subscapularis tear (any)	10	3 (5)	7 (5)	0.8 (0.2, 3.3)	1.000
- PTT	4	1 (2)	3 (2)	0.7 (0.1, 6.4)	1.000
- FTT	1	0 (0)	1 (1)	1.5 (1.4, 1.7)	1.000
Supraspinatus tendinosis	27	9 (14)	18 (14)	1.0 (0.4, 2.3)	1.000
Infraspinatus tendinosis	1	0 (0)	1 (1)	1.5 (1.4, 1.7)	1.000
Subscapularis tendinosis	4	0 (0)	4 (3)	1.5 (1.4, 1.7)	0.302
Supraspinatus calcification	33	16 (24)	17 (13)	2.1 (1.0, 4.5)*	0.050
Infraspinatus calcification	9	3 (5)	6 (5)	1.0 (0.2, 4.1)	1.000
Subscapularis calcification	20	5 (8)	15 (12)	0.6 (0.2, 1.8)	0.462
LHB tear or tendinosis	6	2 (3)	4 (3)	1.0 (0.2, 5.5)	1.000
LHB tendon sheath effusion	26	10 (15)	16 (12)	1.3 (0.5, 3.0)	0.657

*Abbreviations*: PAR, positive anaesthetic response; NAR, negative anaesthetic response; OR, unadjusted odds ratio; ACJ, acromioclavicular joint; GHJ, glenohumeral joint; SAB, subacromial bursa; CAL, coracoacromial ligament; PTT, partial thickness tear; FTT, full thickness tear; LHB, long head of biceps.

*Note*: Pathology subgroup totals may exceed composite pathology totals due to some cases identified in which multiple pathologies were present.

^†^ SAB pathology included: thickening ≥2mm, calcification, bursal fluid or effusion.

*p≤0.05

Two hundred and two patients received the reference standard procedure (SAB diagnostic injection) (Fig 3). Two anaesthetic responses were not recorded as one participant was unable to understand VAS scales and one suffered transient post-injection weakness preventing post-injection clinical testing and clinical tests could not be repeated. Four anaesthetic responses were excluded from the analysis for which pre-injection pain intensity was low (<20mm) and this is known to affect the accuracy of post-injection change estimations using VAS scales [59]. This resulted in 196 cases being included in the analysis.

Diagnostic accuracy characteristics for the clinical examination variables are presented in Table 4 (S1 Table). Highest sensitivity (99%) and lowest LR- (0.14) were observed for full passive external rotation (ER). Highest specificity (97%) was observed for external rotation lag sign, and highest LR+ (2.6) for the lack of symptom provocation at end range abduction/external rotation (ABER). A loss of passive ER is the key diagnostic clinical feature for a ‘stiff’ shoulder due to frozen shoulder or glenohumeral arthritis. Cases with restriction of passive ER greater than 30° compared with the unaffected side (n = 15) were therefore excluded from further analysis to avoid the inclusion of competing clinical entities that would be managed differently in the clinical setting. Those with loss of ER who were excluded from further analysis were less likely to report a SAB PAR (OR 7.3; 7.3, 95%CI 0.9, 57.0) and had a higher prevalence of GHJ arthropathy on x-ray compared to those with full passive ER (17% vs 3% respectively) (OR 7.2, 95%CI 1.6, 33.1; p = 0.025). There were no other differences in demographic characteristics or other imaging features in those who were excluded from analysis due to a loss of passive ER (p>0.05).

**Table 4:** Diagnostic accuracy of clinical examination variables for a positive response to diagnostic injection into the subacromial bursa. (N = 196).

Clinical examination variables	Sens (95% CI)	Spec (95% CI)	PPV (95% CI)	NPV (95% CI)	LR+ (95% CI)	LR- (95% CI)	DOR (95% CI)
Age > 50 yrs	29 (19, 41)	69 (60, 76)	32 (21, 44)	65 (57, 73)	0.91 (0.57, 1.42)	1.04 (0.84, 1.25)	0.9 (0.5, 1.7)
Traumatic onset^†^	26 (17, 37)	56 (48, 64)	23 (15, 34)	60 (51, 68)	0.59 (0.37, 0.90)	1.32 (1.06, 1.63)	0.4 (0.2, 0.9)
Strain injury^†^	55 (43, 66)	65 (57, 73)	44 (34, 55)	74 (65, 81)	1.58 (1.13, 2.17)	0.70 (0.51, 0.91)	2.3 (1.3, 4.1)
Lateral shoulder pain^†^	20 (12, 31)	80 (72, 86)	33 (21, 49)	66 (59, 73)	0.99 (0.54, 1.75)	1.00 (0.85, 1.15)	1.0 (0.5, 2.1)
Anterior shoulder pain^†^	42 (31, 54)	73 (65, 80)	44 (33, 57)	71 (63, 78)	1.58 (1.05, 2.33)	0.79 (0.61, 0.98)	2.0 (1.1, 3.7)
Night pain disturbs sleep	58 (46, 69)	51 (42, 59)	37 (28, 47)	71 (61, 79)	1.18 (0.88, 1.53)	0.83 (0.59, 1.14)	1.4 (0.8, 2.6)
Painful arc in abduction	56 (43, 67)	39 (30, 48)	35 (26, 44)	60 (48, 71)	0.91 (0.68, 1.17)	1.14 (0.79, 1.63)	0.8 (0.4, 1.5)
Resisted abd. or ER pain	76 (64, 85)	18 (12, 25)	33 (26, 40)	58 (42, 72)	0.92 (0.77, 1.06)	1.39 (0.78, 2.42)	0.7 (0.3, 1.4)
Resisted int rotation pain	50 (38, 62)	52 (44, 61)	36 (27, 46)	67 (57, 75)	1.05 (0.77, 1.41)	0.96 (0.70, 1.26)	1.1 (0.6, 2.0)
Full passive ER (0°)^ǂ^	99 (92, 100)	11 (7, 17)	36 (30, 43)	93 (70, 99)	1.11 (1.02, 1.20)	0.14 (0.02, 0.79)	7.3 (0.9, 57.0)
No end-range pain passive ER (90°)	40 (29, 52)	84 (77, 90)	57 (42, 70)	74 (66, 80)	2.56 (1.56, 4.21)	0.71 (0.56, 0.86)	3.6 (1.8, 7.2)
Full passive ER (90°)^ǂ^	92 (84, 97)	18 (13, 26)	37 (30, 45)	82 (64, 92)	1.13 (1.00, 1.26)	0.42 (0.17, 0.99)	2.7 (1.0, 7.5)
Hawkins-Kennedy test +	59 (46, 70)	29 (22, 38)	30 (23, 39)	57 (45, 69)	0.83 (0.64, 1.03)	1.42 (0.95, 2.10)	0.6 (0.3, 1.1)
Drop arm +	12 (6, 22)	90 (84, 94)	40 (22, 61)	66 (59, 73)	1.26 (0.55, 2.84)	0.97 (0.85, 1.07)	1.3 (0.5, 3.4)
Empty Can test + (pain or weakness)	86 (76, 93)	14 (9, 21)	35 (28, 43)	65 (46, 81)	1.00 (0.87, 1.12)	0.99 (0.47, 2.03)	1.0 (0.4, 2.4)
ERLS +	5 (2, 13)	97 (92, 99)	43 (16, 75)	66 (59, 73)	1.44 (0.37, 5.59)	0.99 (0.90, 1.05)	1.5 (0.3, 6.7)

*Abbreviations*: Sens, sensitivity; CI, confidence interval; Spec, specificity; PPV, positive predictive value; NPV, negative predictive value; +LR, positive likelihood ratio; -LR, negative likelihood ratio; DOR, diagnostic odds ratio; ER, external rotation; ER(0°), external rotation performed in neutral; ER(90°), external rotation performed at 90° abduction; +, positive test result; ERLS, external rotation lag sign.

^†^ Operational definitions for history variables (onset and pain location) are provided in a supplementary file (S1 Table).

^ǂ^ Full passive external rotation defined by less than 30° deficit compared with the unaffected side.

Diagnostic accuracy characteristics for the imaging variables with the highest odds (>2.0) of a PAR (supraspinatus calcification on x-ray, and supraspinatus calcification and a full-thickness supraspinatus tear (FTT) on ultrasound) are presented in Table 5 (S2 Table). A supraspinatus tear on ultrasound demonstrated the highest LR+ (6.2) regardless of age group and highest specificity for a PAR (100%) in the under-50 age group.

**Table 5:** Diagnostic accuracy of imaging variables for a positive response to diagnostic injection into the subacromial bursa.

Imaging Findings	Sens (95% CI)	Spec (95% CI)	PPV (95% CI)	NPV (95% CI)	LR+ (95% CI)	LR- (95% CI)	DOR (95% CI)
**All participants (n = 180)**							
SSp calcium (XR or USS)	25 (16, 36)	86 (79, 91)	50 (34, 66)	67 (59, 74)	1.77 (0.95, 3.26)	0.88 (0.73, 1.01)	2.0 (0.9, 4.4)
SSp calcium (XR)	14 (8, 25)	95 (89, 98)	60 (36, 80)	67 (59, 73)	2.70 (1.04, 6.97)	0.91 (0.79, 1.00)	3.0 (1.0, 8.8)
SSp calcium (USS)	23 (15, 35)	87 (80, 92)	50 (33, 67)	67 (59, 74)	1.77 (0.93, 3.34)	0.89 (0.74, 1.02)	2.0 (0.9, 4.4)
SSp FTT (USS)	11 (5, 21)	98 (94, 100)	78 (45, 94)	66 (59, 73)	6.19 (1.51, 25.69)	0.91 (0.81, 0.98)	6.8 (1.4, 33.9)
**Age ≥ 50 yrs (n = 49)**							
SSp calcium (XR or USS)	33 (16, 56)	81 (64, 91)	50 (25, 75)	68 (52, 81)	1.72 (0.66, 4.38)	0.83 (0.53, 1.15)	2.1 (0.6, 7.80
SSp calcium (XR)	11 (3, 33)	94 (79, 98)	50 (15, 85)	64 (50, 77)	1.72 (0.32, 9.09)	0.95 (0.71, 1.15)	1.8 (0.2, 14.1)
SSp calc (USS)	33 (16, 56)	84 (67, 93)	55 (28, 79)	68 (53, 81)	2.07 (0.75, 5.60)	0.80 (0.51, 1.09)	2.6 (0.7, 10.2)
SSp FTT (USS)	28 (13, 51)	94 (79, 98)	71 (36, 92)	69 (54, 81)	4.31 (1.06, 17.90)	0.77 (0.52, 0.99)	5.6 (1.0, 32.6)
**Age < 50 yrs (n = 131)**							
SSp calcium (XR or USS)	21 (12, 35)	88 (80, 93)	50 (30, 70)	67 (58, 75)	1.79 (0.81, 3.89)	0.89 (0.73, 1.04)	2.0 (0.8, 5.2)
SSp calcium (XR)	15 (8, 28)	95 (88, 98)	64 (35, 85)	67 (58, 75)	3.20 (1.05, 9.77)	0.89 (0.75, 1.00)	3.6 (1.0, 13.0)
SSp calc (USS)	19 (10, 33)	88 (80, 93)	47 (27. 68)	66 (57, 74)	1.61 (0.71, 3.58)	0.92 (0.76, 1.06)	1.8 (0.7, 4.7)
SSp FTT (USS)	4 (1, 14)	100 (96, 100)	100 (34, 100)	65 (57, 73)	∞ (0.95, ∞)	0.96 (0.99, ∞)	2.9 (2.3, 3.6)

*Abbreviations*: Sens, sensitivity; CI, confidence interval; Spec, specificity; PPV, positive predictive value; NPV, negative predictive value, LR+, positive likelihood ratio; LR-, negative likelihood ratio; DOR, diagnostic odds ratio; SSp, supraspinatus; XR, x-ray; USS, diagnostic ultrasound scan; FTT, full thickness tear.

Where LR include infinity, values for predictive values represent estimated post-test probability and 95% CI.

**Fig 4:**
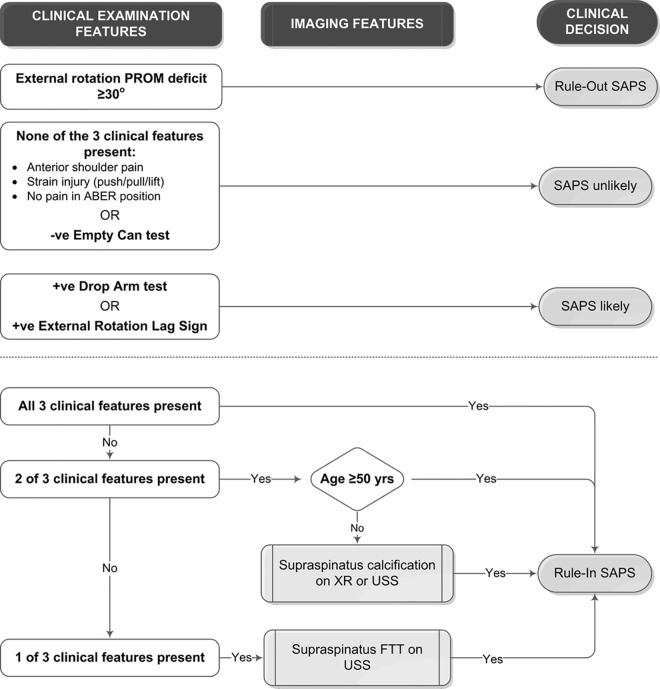
Diagnostic algorithm. Algorithm for identifying participants with subacromial pain defined by ≥80% post-injection pain relief following ultrasound guided injection of local anaesthetic into the subacromial bursa. *Abbreviations*: PROM, passive range of motion; SAP, subacromial pain; ABER, abduction/external rotation (external rotation performed at 90o abduction); -ve, negative; +ve, positive; XR, x-ray; USS, diagnostic ultrasound scan; FTT, full thickness tear

Three clinical variables were identified with the highest LR+ and DOR for an 80% PAR for which the lower confidence limits exceeded 1.0: strain injury, anterior shoulder pain and the inability to reproduce symptoms at end-range abduction/external rotation (ABER). These variables were combined and diagnostic characteristics recalculated (Table 6. Data in S3 Table). For combinations of clinical examination and imaging variables, highest sensitivity (85%) and lowest LR- (0.43) were observed when none of the clinical features were present for both age groups. Highest specificity (100) and LR+ (infinity) were observed when all three clinical features were present in both age groups. In the over-50 age group, two or more clinical features combined with supraspinatus calcium deposits on x-ray or USS yielded the highest specificity (100) and LR+ (infinity) for an 80% PAR. In the under-50 age group, one or more clinical features combined with supraspinatus FTT on USS yielded the highest specificity (100) and LR+ (infinity) for an 80% PAR. Sensitivity was low (<29%) for all combinations of clinical examination and imaging variables in both age groups. A diagnostic flow chart was developed providing possible clinical applications of the results. (Fig 4).

**Table 6:** Diagnostic accuracy of combinations of clinical examination variables alone, and when combined with diagnostic ultrasound scan reports of supraspinatus pathology.

Combinations of Clinical Variables	Sens (95% CI)	Spec (95% CI)	PPV (95% CI)	NPV (95% CI)	LR+ (95% CI)	LR- (95% CI)	DOR (95% CI)
**All Participants (n = 180)**							
1 clinical feature^†^	85 (74, 91)	36 (28, 45)	43 (34, 51)	80 (68, 89)	1.32 (1.10, 1.57)	0.43 (0.23, 0.77)	3.0 (1.4, 6.6)
2 clinical features^†^	45 (33, 57)	85 (77, 91)	63 (49, 76)	73 (65, 90)	3.00 (1.80, 5.00)	0.65 (0.50, 0.80)	4.6 (2.3, 9.4)
3 clinical features^†^	9 (4, 19)	100 (97, 100)	100 (61, 100)	67 (59, 73)	∞ (2.87, ∞)	0.91 (0.94, ∞)	3.0 (2.4, 3.7)
**Age ≥50 years (n = 49)**							
1 of 3 clinical features:^†^	83 (61, 94)	39 (24, 61)	44 (29, 61)	80 (55, 93)	1.36 (0.93, 1.97)	0.43 (0.14, 1.17)	(0.8, 13.3)
- With SSp calcium on XR or USS	28 (13, 51)	94 (79, 98)	71 (36, 92)	69 (54, 81)	4.31 (1.06, 17.90)	0.77 (0.52, 0.99)	(1.0, 32.6)
- With SSp FTT on USS	28 (13, 51)	94 (79, 98)	71 (36, 92)	69 (54, 81)	4.31 (1.06, 17.90)	0.77 (0.52, 0.99)	5.6 (1.0, 32.6)
2 of 3 clinical features:^†^	44 (25, 66)	97 (84, 99)	89 (57, 98)	75 (60, 86)	13.78 (2.51, 81.36)	0.57 (0.35, 0.79)	(2.7, 216.3)
- With SSp calcium on XR or USS	22 (9, 45)	100 (89, 100)	100 (51, 100)	69 (54, 81)	∞ (1.91, ∞)	0.78 (0.89, ∞)	(2.1, 5.0)
- With SSp FTT on USS^‡^							
3 clinical features:^†^	6 (1, 26)	100 (89, 100)	100 (21, 100)	64 (50, 77)	^§^	^§^	(2.0, 4.1)
- With SSp calcium on XR or USS	6 (1, 26)	100 (89, 100)	100 (21, 100)	64 (50, 77)	^§^	^§^	(2.0, 4.1)
- With SSp FTT on USS^‡^							
**Age <50 years (n = 131)**							
1 of 3 clinical features:^†^	85 (72, 93)	35 (25, 45)	42 (33, 52)	81 (65, 90)	1.30 (1.06, 1.59)	0.43 (0.20, 0.87)	(1.2, 7.6)
- With SSp calcium on XR or USS	21 (12, 35)	93 (85, 97)	63 (39, 82)	68 (59, 76)	2.98 (1.19, 7.45)	0.85 (0.70, 0.97)	(1.2, 10.4)
- With SSp FTT on USS	4 (1, 14)	100 (96, 100)	100 (34, 100)	65 (57, 73)	∞ (0.95, ∞)	0.96 (0.99, ∞)	2.9 (2.3, 3.6)
2 of 3 clinical features:^†^	45 (31, 59)	81 (71, 88)	57 (41, 71)	72 (62, 80)	2.32 (1.35, 3.98)	0.69 (0.50, 0.88)	(1.5, 7.5)
- With SSp calcium on XR or USS	15 (7, 28)	98 (92, 99)	78 (45, 94)	67 (58, 75)	6.18 (1.52, 25.45)	0.87 (0.74, 0.96)	(1.4, 35.7)
- With SSp FTT on USS^‡^							
3 clinical features:^†^	11 (5, 23)	100 (96, 100)	100 (57, 100)	67 (59, 75)	∞ (2.44, ∞)	0.89 (0.94, ∞)	(2.4, 3.9)
- With SSp calcium on XR or USS	^§^	^§^	^§^	^§^	^§^	^§^	(2.3, 3.7)
- With SSp FTT on USS^‡^							

*Abbreviations*: Sens, sensitivity; Spec, specificity; PPV, positive predictive value; NPV, negative predictive value, LR+, positive likelihood ratio; LR-, negative likelihood ratio; DOR, diagnostic odds ratio; SSp, supraspinatus; XR, plain x-ray; USS, diagnostic ultrasound scan; FTT, full thickness tear.

^**†**^ Represents the minimum number of positive clinical features (strain mechanism of injury, anterior shoulder pain and absence of symptom reproduction with passive range of motion external rotation (at 90° abduction)) required to fit the criterion.

^‡^ No cases identified fitting the criterion, invalid calculation.

^§^ Insufficient cases for valid calculation.

Where LR include infinity, values for predictive values represent estimated post-test probability and 95% CI.

## Discussion

In patients with no significant capsular restriction of the shoulder, combinations of three clinical examination features (anterior shoulder pain, strain injury and the absence of symptoms at end range abduction/external rotation (ABER)) helped confirm the presence of subacromial pain, defined by ≥80% pain relief following an injection of local anaesthetic into the subacromial bursa. The majority of imaging features were of limited diagnostic value, with the exception of supraspinatus pathology (calcification or full thickness tear) which increased the likelihood of SAP in this cohort irrespective of age. No individual or combinations of clinical examination and imaging findings were able to rule-out SAP with high levels of confidence.

A loss of passive external rotation is the key diagnostic feature for conditions causing capsular restriction of the glenohumeral joint including frozen shoulder and arthritis [47]. For this reason, full passive range of motion is reported as a precondition for the diagnosis of a rotator cuff tear [42, 60]. Our findings support this clinical construct indicating that those with more than 30° restriction of external rotation (performed in neutral) were 7 times less likely to have SAP (sensitivity 99%) and had a higher prevalence of glenohumeral joint arthropathy on x-ray. Confidence in this result is high, with narrow confidence limits for sensitivity and the negative likelihood ratio. To our knowledge, this is the first report of the diagnostic accuracy of “full passive external rotation” for painful subacromial conditions.

### Clinical examination variables

The clinical examination variables that produced the highest likelihood of SAP were anterior shoulder pain, strain injury (push, pull or lifting injury) and the absence of symptom provocation in ABER. These are the same three variables previously identified as the strongest predictors of a PAR using multivariate analyses (adjusted odds ratio 2.3–3.9) [38]. However, the positive likelihood ratios for these variables were 1.6, 1.6 and 2.6 respectively indicating that, when used in isolation, these clinical features result in only a small increase in the likelihood of SAP. However, when used in combination, the three clinical features were able to rule-in a PAR with high levels of confidence when all three were present. In those older than 50 years, two of the three clinical features also provided confidence in identifying SAP. However, there was little change in the post-test probability of SAP when none of the three variables were identified, and the absence of these clinical features would therefore not allow SAP to be ruled-out with confidence.

The inability to reproduce typical symptoms at end range ABER increased the likelihood of SAP in this study. Pain provocation at end range ABER is reported to be indicative of glenohumeral joint pathology including capsulo-labral and articular surface supraspinatus lesions [61, 62]. Although symptom reproduction in this position does not permit identification of a specific pathoanatomic lesion, the absence of symptoms in this position may help exclude the GHJ as a source of pain. The three most common causes of shoulder pain are the GHJ, the acromioclavicular joint and subacromial conditions [5]. The ability to eliminate one or more of these sources of shoulder pain would thereby increase the probability of another (diagnosis by exclusion). Thus, the absence of symptoms at end range ABER may help eliminate the GHJ as a source of pain thereby increasing the likelihood of an alternate source of shoulder pain such as subacromial pain.

Overall, few individual clinical variables yielded highly accurate diagnostic estimates. Accuracy of the empty can test (sensitivity 86%), drop arm sign and external rotation lag signs (specificity 90–97%) was similar in this study compared with previous reports for rotator cuff integrity [16–18, 63, 64]. However, clinical tests previously reported to be diagnostic for subacromial “impingement” pathology including Hawkins-Kennedy test and painful arc in abduction [34] were of limited value for identifying SAP in the current study. Our results differ from others who used a subacromial local anaesthetic injection reference standard, yielding lower sensitivity for the Hawkins-Kennedy test (59% vs 92%) and lower specificity for arc pain in abduction (56% vs 81%). Previous studies reported higher sensitivity (69–88%) and specificity (45–81%) for the Hawkins-Kennedy test and painful arc in abduction for all stages of surgically visualized “impingement” pathology [17, 65]. Possible explanations for the differences in results include previous studies recruiting patients from rheumatology or orthopaedic clinics where the prevalence and severity of painful subacromial conditions is likely to differ from primary health care settings affecting the frequency of positive or negative clinical examination test results [66]. Differences in the reference standard tests (diagnostic injection vs surgically visualized pathology) may also account for differences in results. Some visualized pathology may not have been symptomatic in previous studies leading to a higher number of ‘true positive’ results and increased sensitivity. In our study, some intra-tendinous pathology may not have been exposed to anaesthetic in the presence of an intact bursa/cuff interface increasing the number of false negative results resulting in decreased sensitivity.

### Imaging variables

A full-thickness supraspinatus tear on diagnostic ultrasound scan increased the likelihood of SAP by almost 7 times compared to those with an intact supraspinatus for all participants across all age groups. In the hands of skilled operators using modern equipment, as was the case in this study, the pooled sensitivity and specificity of ultrasound is 92% and 95% respectively, similar to magnetic resonance imaging [67]. In agreement with previous reports, full-thickness supraspinatus tears were more common in those over 50 years in our study (11% vs 2% in the under-50 age group) [29, 30, 68, 69]. However, in this study involving symptomatic patients, the presence of a full thickness supraspinatus tear in those older than 50 years resulted in high specificity (94%), LR+ (4.3) and odds (5.6) of a PAR, indicating that many of these tears may in fact be symptomatic. It is well known that the prevalence of supraspinatus tears increases with age in asymptomatic populations [29]. These results provide preliminary indications that full thickness supraspinatus tears may be of symptomatic relevance in symptomatic populations, irrespective of age. Studies involving larger numbers are required to confirm this finding.

Supraspinatus tendon calcification on x-ray or ultrasound scan also increased the probability of SAP in this study. Calcification is a cell-mediated process characterised by asymptomatic stages (formative and resting phase) and a highly symptomatic resorptive phase that most commonly affects those between the ages of 30 and 50 years [70]. Participants with calcification reported on x-ray were 3.0 times more likely to have SAP, and this likelihood increased in those younger than 50 years to 3.6 times. Our results provide an initial indication that calcific lesions may be of symptomatic relevance especially in those <50 years of age.

Pathology affecting the subacromial bursa (fluid, effusion, thickening or calcification) and bunching of the subacromial bursa under the lateral edge of the acromion was reported in approximately 40% of patients with shoulder pain in this cohort [71]. These findings were not associated with a PAR at the 80% threshold (p>0.05) and did not increase the odds of a PAR when present in this study. Care is therefore required when interpreting this finding with respect to the use of targeted pain relief interventions such as therapeutic corticosteroid injections in the clinical setting.

### Combinations of clinical examination and imaging findings

When combined with the clinical examination findings, imaging features improved the ability to rule-in, but not to rule-out SAP. When a supraspinatus FTT or calcification was combined with one of the three clinical features a marked increase in specificity (94%) and post-test probability (71%) for a PAR was observed in both age groups. In the under-50 age group, the low prevalence of FTT affected the precision of diagnostic estimates and results should be interpreted accordingly in the clinical setting.

The x-ray or USS report of calcific deposits in supraspinatus also increased the likelihood of SAP by more than 5 times when combined with at least one clinical feature in the over-50 age group, and by more than 7 times when combined with two clinical features in the under-50 age group. We are not aware of any other studies in which the accuracy of combinations of clinical examination and imaging features have been combined using diagnostic injection as the reference standard test.

There were some limitations to consider. The false-positive rate for subacromial bursa diagnostic injection procedures is unknown, however all possible steps were taken to ensure accuracy of needle placement using imaging guidance. Analysis of post-injection pain response was undertaken according to international guidelines that recommend exclusion of cases from analysis with low pre-injection pain severity that may increase the rate of false positive or negative results.

In patients with no significant GHJ capsular restriction, combinations of patient history and physical examination features helped positively identify subacromial pain. The presence of supraspinatus calcification or a full thickness tear on ultrasound scan increased the likelihood of SAP regardless of age indicating these variables are likely to be of symptomatic relevance in the primary care setting.

## Supporting Information

S1 Table Clinical Examination Data and Operational Definitions. Operational definitions and contingency cell counts for clinical examination variables for a positive response to diagnostic injection of local anaesthetic into the subacromial bursa.
(PDF)

S2 Table Imaging Data. Contingency cell counts for imaging variables for a positive response to diagnostic injection of local anaesthetic into the subacromial bursa.
(PDF)

S3 Table Combined Clinical Examination and Imaging Data. Contingency cell counts for combinations of clinical examination variables alone, and when combined with diagnostic ultrasound scan reports of supraspinatus pathology.
(PDF)
