# Supplementary material for: Diagnostic Accuracy of Clinical Examination and Imaging Findings for Identifying Subacromial Pain
Source: PLoS One. 2016 Dec 9;11(12):e0167738. doi: 10.1371/journal.pone.0167738 (PMC5147961; doi:10.1371/journal.pone.0167738)
Supplement: S1 Table — Operational definitions and contingency cell counts for clinical examination variables for a positive response to diagnostic injection of local anaesthetic into the subacromial bursa. (PDF) [file pone.0167738.s001.pdf]

S1 Table:

Contingency cell counts for clinical examination variables for a positive response to diagnostic injection of local anaesthetic into the subacromial bursa. (Table 4 data).

Clinical examination variables	TP	FN	FP	TN
Age > 50 yrs	19	47	41	89
Traumatic onset[a]	17	49	57	73
Strain injury[b]	36	30	45	85
Lateral shoulder pain[c]	13	53	26	104
Anterior shoulder pain[d]	28	38	35	95
Night pain disturbs sleep	37	27	63	65
Supraspinatus atrophy	24	37	44	72
Painful arc in abduction	35	28	66	42
Resisted abd. or ER pain	50	16	104	22
Resisted internal rotation pain	33	33	60	66
Full passive ER (0°)†	65	1	116	14
No end-range pain passive ER (90°)	26	39	20	108
Full passive ER (90°)†	61	5	103	23
Hawkins-Kennedy test +	38	27	87	36
Drop arm +	8	58	12	113
Empty Can test + (pain or weakness)	57	9	106	17
ERLS +	3	63	4	123

Abbreviations. TP, true positives; FN, false negative; FP, false positive; TN, true negatives; abd, abduction; ER, external rotation; ER, external rotation; ER(0°), external rotation performed in neutral; ER(90°), external rotation performed at 90° abduction; +, positive test result; ERLS, external rotation lag sign.

[a-d]See table of operational definitions below.

† Full passive external rotation defined by less than 30° deficit compared with the unaffected side.

Note: Total cell counts are less than 196 for some variables due to missing data.

Operational Definitions for History Variables	
[a]Traumatic onset	Fall or high force impact through upper limb, or directly to shoulder region.
[b]Strain injury	Lifting, reaching with load, push, pull, throwing motion.
[c]Lateral shoulder pain	Pain located between the acromion (superiorly) and deltoid insertion (inferiorly).
[d]Anterior shoulder pain	Pain located between the coracoid process (medially) and the anterior edge of the acromion (laterally) and bounded by the AC joint superiorly.
Superior shoulder pain	Pain located along the borders of the clavicle (anteriorly), spine of scapula (posteriorly) and bounded by the AC joint (laterally).
Posterior shoulder pain	Pain located posteriorly, medial to the deltoid insertion and inferior to the spine of the scapula.
