# Supplementary material for: Diagnostic Accuracy of Clinical Examination and Imaging Findings for Identifying Subacromial Pain
Source: PLoS One. 2016 Dec 9;11(12):e0167738. doi: 10.1371/journal.pone.0167738 (PMC5147961; doi:10.1371/journal.pone.0167738)
Supplement: S2 Table — Contingency cell counts for imaging variables for a positive response to diagnostic injection of local anaesthetic into the subacromial bursa. (PDF) [file pone.0167738.s002.pdf]

S2 Table:

Contingency cell counts for imaging variables for a positive response to diagnostic injection of local anesthetic into the subacromial bursa. (For Table 4 in manuscript)

Imaging variables	TP	FN	FP	TN
**All participants (n=180)**				
SSp calcium (XR or USS)	16	49	16	99
SSp calcium (XR)	9	55	6	109
SSp calcium (USS)	15	50	15	100
SSp FTT (USS)	7	58	2	113
**Age ≥ 50 yrs (n= 49)**				
SSp calcium (XR or USS)	6	12	6	25
SSp calcium (XR)	2	16	2	29
SSp calc (USS)	6	12	5	26
SSp FTT (USS)	5	13	2	29
**Age < 50 yrs (n=131)**				
SSp calcium (XR or USS)	10	37	10	74
SSp calcium (XR)	7	39	4	80
SSp calc (USS)	9	38	10	74
SSp FTT (USS)	2	45	0	84

Abbreviations: TP, true positives; FN, false negatives; FP, false positives; TN, true negatives; SSp, supraspinatus; XR, x-ray; USS, diagnostic ultrasound scan; FTT, full thickness tear.

Note: contingency cell counts may not total 180 due to missing data.
