# Supplementary material for: Diagnostic Accuracy of Clinical Examination and Imaging Findings for Identifying Subacromial Pain
Source: PLoS One. 2016 Dec 9;11(12):e0167738. doi: 10.1371/journal.pone.0167738 (PMC5147961; doi:10.1371/journal.pone.0167738)
Supplement: S3 Table — Contingency cell counts for combinations of clinical examination variables alone, and when combined with diagnostic ultrasound scan reports of supraspinatus pathology. (PDF) [file pone.0167738.s003.pdf]

S3 Table:

Contingency cell counts for combinations of clinical examination variables alone, and when combined with diagnostic ultrasound scan reports of supraspinatus pathology. (For Table 5 in manuscript)

Combinations of Clinical Variables	TP	FN	FP	TN
**All Participants (n=180)**				
1 clinical feature†	55	10	74	41
2 clinical features†	29	36	17	97
3 clinical features†	6	58	0	115
**Age ≥50 years (n=49)**				
1 of 3 clinical features:†	15	3	19	12
With SSp calcium on XR or USS	5	13	2	29
With SSp FTT on USS	5	13	2	29
2 of 3 clinical features:†	8	10	1	30
With SSp calcium on XR or USS	4	14	0	31
With SSp FTT on USS‡	0	18	0	31
3 clinical features:†	1	17	0	31
With SSp calcium on XR or USS	1	17	0	31
With SSp FTT on USS‡	0	18	0	31
**Age <50 years (n=131)**				
1 of 3 clinical features:†	40	7	55	29
With SSp calcium on XR or USS	10	37	6	78
With SSp FTT on USS	2	45	0	84
2 of 3 clinical features:†	21	26	16	67
With SSp calcium on XR or USS	7	40	2	81
With SSp FTT on USS‡	0	47	0	83
3 clinical features:†	5	41	0	84
With SSp calcium on XR or USS	2	44	0	84
With SSp FTT on USS‡	0	46	0	84

Abbreviations: TP, true positives; FN, false negatives; FP, false positives; TN, true negatives; SSp, supraspinatus; XR, plain x-ray; USS, diagnostic ultrasound scan; FTT, full thickness tear.

† Represents the minimum number of positive clinical features (strain mechanism of injury, anterior shoulder pain and absence of symptom reproduction with passive range of motion external rotation (at 90° abduction)) required to fit the criterion.

‡ no cases identified fitting the criterion, unable to calculate diagnostic accuracy statistics.

Note: contingency cell counts may not total 180 due to missing data.
